# Hypodermic Use of Morphia during Anæsthesia

**Published:** 1868-08

**Authors:** 


### EDITORIAL DEPARTMENT.

*Hypodermic Use of Morphia during Anæsthesia.*—In the July number of the *Journal* we referred to the effect of alcoholic stimulants upon the action of anæsthetic agents. The following remarks of Prof. W. W. Green, of the Maine Medical School, in a recent clinical lecture in connection with the operation of lithotomy, on the hypodermic use of morphia during anæsthesia, and published in the *Medical Gazette*, may prove interesting to our readers :

" I wish particularly to impress upon your minds the great importance which I attach to two points in connection with the operation just performed, and which you have seen me illustrate repeatedly in other severe operations.

"The first is the use of hot water, or water fully up to blood heat for sponging. I am thoroughly convinced that the use of it instead of the usual cold sponging, diminishes very much the shock, and also the liability to inflammation. My endeavor is to keep the part as near its natural temperature as possible during the operation. The cases where this indication is overbalanced by the necessity of the styptic effect of cold are comparatively rare.

"The second point is the subcutaneous injection of morphine *while the patient is under the full influence of ether* .The influence of this early and speedy introduction of an anodyne into the circulation, in anticipating all pain and irritation and preventing shock, can hardly be over-estimated, especially after severe procedures, and in feeble subjects. But another effect, of the greatest consequence, as regards at least the comfort of the patient and the convenience of all parties, is the decided effect of morphine thus introduced in shortening the anæsthetic influence *and in preventing delirium and nausea.* The gentleman who had the operation for removal of a morbid growth from the rectum, illustrated this effect well. He had twice taken ether before I saw him, and both times was a long while in coming out of its influence, and suffered much from nausea and vomiting. He has been twice etherized under my direction, once for examination, and again for the operation. Both times, while fully under its influence, he got by subcutaneous injection one half grain of morphine. The effect of the anæsthetic rapidly passed away in each instance, with scarcely a trace of delirium and *no nausea.* How it acts I am not certain, but I think by promoting the contractility of the intra-cranial vessels, and so lessening the amount of blood in the brain.‡ This is one of the most important effects of opium in proper doses. I am in the habit of giving a *full* dose; usually not less than a half and often a whole grain. I am quite sure that a trial of this practice is all that is necessary to secure its adoption by surgeons everywhere. My experience thus far indicates that its effect in controlling the nausea following the anæsthetic is more marked in adults than in children."

"‡We think that Professor Greene is in error here. Anæsthetics act, when their influence is fully established, by lessening the supply of blood to the brain, as has been shown by some recent experiments of Dr. Wm. A. Hammond Opium operates in such cases by *increasing* the cerebral circulation.—Ed."

